# Prospective, multicenter validation of the deep learning-based cardiac arrest risk management system for predicting in-hospital cardiac arrest or unplanned intensive care unit transfer in patients admitted to general wards

**DOI:** 10.1186/s13054-023-04609-0

**Published:** 2023-09-05

**Authors:** Kyung-Jae Cho, Jung Soo Kim, Dong Hyun Lee, Sang‑Min Lee, Myung Jin Song, Sung Yoon Lim, Young-Jae Cho, You Hwan Jo, Yunseob Shin, Yeon Joo Lee

**Affiliations:** 1 VUNO, Seoul, Republic of Korea; 2 Division of Critical Care Medicine, Department of Hospital Medicine, Inha College of Medicine, Incheon, Republic of Korea; 3 Department of Intensive Care Medicine, Dong-A University Hospital, College of Medicine, Busan, Republic of Korea; 4 Department of Critical Care Medicine, Seoul National University Hospital, Seoul, Republic of Korea; 5 Division of Pulmonary and Critical Care Medicine, Department of Internal Medicine, Seoul National University Hospital, Seoul National University College of Medicine, Seoul, Republic of Korea; 6 Division of Pulmonary and Critical Care Medicine, Department of Internal Medicine, Seoul National University College of Medicine, Seoul National University Bundang Hospital, Seongnam, Republic of Korea; 7 Department of Emergency Medicine, Seoul National University College of Medicine, Seoul National University Bundang Hospital, Seongnam, Republic of Korea

**Keywords:** Cardiac arrest, Intensive care unit, Early warning score, Artificial intelligence, Deep learning, Rapid response system

## Abstract

**Background:**

Retrospective studies have demonstrated that the deep learning-based cardiac arrest risk management system (DeepCARS™) is superior to the conventional methods in predicting in-hospital cardiac arrest (IHCA). This prospective study aimed to investigate the predictive accuracy of the DeepCARS™ for IHCA or unplanned intensive care unit transfer (UIT) among general ward patients, compared with that of conventional methods in real-world practice.

**Methods:**

This prospective, multicenter cohort study was conducted at four teaching hospitals in South Korea. All adult patients admitted to general wards during the 3-month study period were included. The primary outcome was predictive accuracy for the occurrence of IHCA or UIT within 24 h of the alarm being triggered. Area under the receiver operating characteristic curve (AUROC) values were used to compare the DeepCARS™ with the modified early warning score (MEWS), national early warning Score (NEWS), and single-parameter track-and-trigger systems.

**Results:**

Among 55,083 patients, the incidence rates of IHCA and UIT were 0.90 and 6.44 per 1,000 admissions, respectively. In terms of the composite outcome, the AUROC for the DeepCARS™ was superior to those for the MEWS and NEWS (0.869 vs. 0.756/0.767). At the same sensitivity level of the cutoff values, the mean alarm counts per day per 1,000 beds were significantly reduced for the DeepCARS™, and the rate of appropriate alarms was higher when using the DeepCARS™ than when using conventional systems.

**Conclusion:**

The DeepCARS™ predicts IHCA and UIT more accurately and efficiently than conventional methods. Thus, the DeepCARS™ may be an effective screening tool for detecting clinical deterioration in real-world clinical practice.

*Trial registration* This study was registered at ClinicalTrials.gov (NCT04951973) on June 30, 2021.

**Supplementary Information:**

The online version contains supplementary material available at 10.1186/s13054-023-04609-0.

## Background

Rapid response systems (RRS) have been shown to prevent in-hospital cardiac arrest (IHCA) or unplanned intensive care unit transfer (UIT) by enabling early detection and proper intervention in patients exhibiting signs of clinical deterioration [1, 2]. Track-and-trigger systems are part of the afferent limb of the RRS for monitoring patients, detecting deterioration, and activating the RRS [3]. In general, these can be categorized as single- (SPTTS) or multiple-parameter track-and-trigger systems (MPTTS). SPTTS activate the RRS using single abnormal vital signs or laboratory findings. However, while these systems can be intuitive and sensitive, the rapid response team (RRT) can be exhausted by many false alarms [4]. Early warning scores (EWS) derived from a combination of several physiological parameters are typical examples of MPTTS [5]. The modified early warning score (MEWS) and national early warning score (NEWS) are the most widely used MPTTS [6], both of which have better predictive values for IHCA and are more efficient in detecting clinical deterioration than SPTTS [7, 8].

The deep learning-based cardiac arrest risk management system (DeepCARS™) was first developed in 2018 and approved as a medical device in 2021 by the Ministry of Food and Drug Safety (MFDS). Using basic vital signs (blood pressure [BP], heart rate [HR], body temperature [BT], respiratory rate [RR]), patient age, and the recorded time of each vital sign, the DeepCARS™ has demonstrated higher accuracy in predicting IHCA, compared with the MEWS, with higher sensitivity and a lower false alarm rate [7, 8]. However, the value and safety of this system in real-world practice remain to be determined, given that previous validation studies have been retrospective.

Therefore, we aimed to investigate the predictive accuracy of the DeepCARS™ for IHCA or UIT in general ward patients, compared with that of conventional methods in real-world practice.

## Methods

### Study design and population

We conducted a prospective multicenter cohort study over 3 months (October 18, 2021–January 17, 2022) at four tertiary academic hospitals in South Korea: Inha University Hospital (925 beds), Seoul National University Bundang Hospital (1324 beds), Dong-A University Medical Center (999 beds), and Seoul National University Hospital (1,793 beds). All hospitals had been operating mature RRS for at least 5 years. This study was registered at ClinicalTrials.gov (NCT 04951973) on June 30, 2021. The RRS of each hospital screened and monitored patients with simultaneous running of the DeepCARS™, MEWS, NEWS, and SPTTS for 3 months, and the intervention was maintained as routine practice as originally done by the RRT. As vital signs or laboratory data were entered into the electronic medical record, the prediction score for each method was automatically computed. When an alarm was triggered by any of above methods, the RRT reviewed and confirmed the alarm, making a decision on whether to provide intervention. It is important to note that the alarms generated by each method did not require any mandatory action, as it primarily serves as a screening tool.

All patients aged 18 years who had been admitted to the general wards during the study period were included. Patient data were excluded in the following cases: admission date outside of the study period, admission within 24 h before the end of the study period among those who did not experience IHCA or UIT, no vital signs recorded 24 h before IHCA or UIT, no vital signs recorded during the entire study period, and patients with DNR orders without any occurred events (Additional file 1: Fig. S1). The Ethics Committee and Institutional Review Board of each hospital approved the study protocol as minimal-risk research using data collected for routine clinical practice, and they waived the requirement of informed consent.

### Outcomes

The primary outcome of interest was the composite of IHCA (loss of circulation prompting resuscitation with chest compression, defibrillation, or both) and UIT (admission to the intensive care unit (ICU) due to unanticipated deterioration in patients from general wards rather than from the operating room or emergency department) [9–11]. We compared the predictive accuracy of the DeepCARS™ with that of the conventional triggering systems (MEWS, NEWS, and SPTTS) to determine whether the primary outcome occurred within 24 h of the system alarm being triggered. Additionally, we compared each score in terms of alarm performance and the timeliness of prediction. In addition, subgroup analyses were conducted according to department of admission, age group, sex, hospital, and surgical status.

### Data collection and preprocessing

We collected data on age, sex, occurrence of events (IHCA and UIT), recorded time of vital signs, five time-stamped vital sign values (BP [systolic and diastolic], HR, RR, and BT), consciousness level, oxygen saturation, oxygen supplementation, five time-stamped laboratory test values (pH, PaO_2_, PaCO_2_, TCO_2_, and lactic acid), scores derived using each triggering system, DNR code status, and RRT intervention.

### Deep learning-based cardiac arrest risk score

The detailed architecture of the DeepCARS™ has been described previously [7, 8].

### Deployment of the DeepCARS™

We deployed the DeepCARS™ and dashboard software in all participating hospitals. The design and interface choices for the dashboard were made in collaboration with the RRT from all participating hospitals and were refined based on the initial draft. The deployment was conducted in two steps. First, the RRT from the site and development team of the DeepCARS™ met with clinicians and the information system team to explain the features of the system, share the integration specifications, and discuss how to integrate the product within the hospital. Next, we set up the implementation phase to verify system integration at each site. The dashboard was used to display alerts and values for each prediction model and record the final intervention performed. We designed a dashboard for the RRT to click a button to categorize alerts into four types of events: cardiopulmonary resuscitation (CPR), UIT, DNR suggestion, and borderline intervention. Alerts that occurred in all hospitals after activation were included in the analysis.

### Performance evaluation and statistical analysis

#### Key aspect 1: How accurate is the DeepCARS™ in predicting IHCA or UIT, compared with conventional methods?

We evaluated predictive performance by measuring the area under the receiver operating characteristic curve (AUROC), which is one of the most used metrics reflecting sensitivity/false positive rates. Additionally, we calculated the F-1 score (2 × [precision × recall]/[precision + recall]), positive predictive value (true positive/[true positive + false positive]), negative predictive value (true negative/[true negative + false negative]), net reclassification index, and number needed to examine (NNE) [12, 13]. We also compared predictive performance according to the timeline in the prediction window (24, 12, 6, 3, and 0.5 h before the primary event).

#### Key aspect 2: Does the DeepCARS™ lead to a lower total alarm count and higher appropriate alarm rate, compared with conventional methods?

We compared alarm performance by measuring the total alarm count and the rate of appropriate alarms. The total alarm count was expressed as the mean alarm count per day (MACPD)/1,000 beds and calculated by dividing the total number of alarms by the study period and the total number of beds and multiplying it by 1,000. Lower MACPD indicates better alarm performance.

We triaged the interventions performed by the RRT according to the A/B/C categories used by critical care response teams in Ontario [14], with minor modifications. We divided patients into the following four categories: Category A (admission to the ICU); category B (borderline) included patients who required further assessment (typically investigations or monitoring of response to therapy); and category Cp (CPR) included patients with loss of circulation, prompting resuscitation with chest compression, defibrillation, or both. We added category D (do not resuscitate [DNR]), which included patients whose DNR orders were initiated by the RRT in the ward [15]. All other alarms were categorized as Z. An alarm that activated the RRT and was connected to clinical intervention categories A, B, C, and D was defined as an appropriate alarm.

The rate of appropriate alarms was calculated by dividing the number of appropriate alarms by the total alarm count as follows: we compared the appropriate alarm count at MEWS and NEWS values of 5 points, which is the most commonly used triggering threshold and equivalent to a score of 95 points for the DeepCARS™.

#### Key aspect 3: Does the DeepCARS™ predict more cases of IHCA or UIT earlier than conventional systems do at the same specificity level?

Delayed RRT intervention is associated with poor prognosis [16]. When there is sufficient preparation time for the RRT before a patient falls into a disastrous condition, the team has the advantage of responding appropriately to the deteriorating patient. Therefore, the ability to predict more events in a timely manner is an important feature of the RRS. We analyzed this performance by comparing the cumulative percentages of patients with composite primary outcomes from 24 h to 0.5 h before the event.

#### Key aspect 4: How robust is the DeepCARS™ in various cohorts when compared with conventional methods?

We calculated the predictive performance of the DeepCARS™ in various cohorts in terms of department of admission. The cohort was also divided according to age, sex, hospital, and surgical status.

Additionally, we assessed the calibration of each DeepCARS™ prediction model by plotting ideal calibration curves and calculating the average absolute error between the actual and estimated outcomes. We performed extensive statistical analysis using scikit-learn (Scikit-learn 0.23.1; community-driven project sponsored by BCG GAMMA), pandas (Pandas 1.0.5; community-driven project sponsored by NumFOCUS), and R (R 3.6.1; R core Team 2021).

## Results

### Baseline characteristics

In total, 55,083 patients admitted to the general wards of four teaching hospitals were included (Additional file 1: Fig. S1). The incidence rate of IHCA in the general wards was 0.90/1,000 admissions, and the rate of UIT was 6.44/1,000 admissions. Borderline intervention and DNR by RRT rates were 15.70/1,000 admissions and 1.01/1,000 admissions, respectively (Table 1).

**Table 1:** Baseline characteristics

Characteristics	Overall cohort	Hospital A	Hospital B	Hospital C	Hospital D	p-value
Number of total admissions, n	55,083	8754	18,214	9,020	19,095	–
Number of observation sets, n	2,855,679	363,765	837,466	254,342	1,400,106	–
Age, y, mean ± SD	60.06 ± 15.95	60.77 ± 16.71	59.76 ± 16.01	62.58 ± 15.22	58.80 ± 15.73	< 0.001
Length of stay, mean ± SD	5.81 ± 11.53	7.02 ± 21.39	4.70 ± 5.61	8.61 ± 14.30	5.00 ± 6.40	< 0.001
Male, sex, n (%)	26,980 (48.98%)	4,448 (50.81%)	8,751 (48.04%)	4,907 (54.40%)	8,874 (46.47%)	< 0.001
Variables within 24 h before outcome (IHCA or UIT) patients, mean ± SD						
SBP (mmHg)	116.17 ± 28.82	117.40 ± 29.89	115.25 ± 27.73	114.19 ± 24.26	117.14 ± 31.54	< 0.05
DBP (mmHg)	68.16 ± 17.02	68.07 ± 17.06	66.90 ± 17.14	69.80 ± 15.38	69.60 ± 17.57	< 0.001
HR (/min)	98.62 ± 23.64	96.93 ± 24.43	98.99 ± 22.46	101.48 ± 25.16	98.83 ± 24.07	< 0.001
RR (/min)	21.69 ± 6.60	20.97 ± 4.81	21.59 ± 7.90	22.61 ± 5.16	22.63 ± 6.67	< 0.001
BT (°C)	37.03 ± 0.95	37.11 ± 1.11	36.97 ± 0.85	36.82 ± 0.63	37.18 ± 0.96	< 0.001
SpO2 (%)	95.72 ± 4.61	96.10 ± 4.65	95.40 ± 4.43	95.44 ± 5.14	96.17 ± 4.63	< 0.001
Lactic acid (mmol/L)	4.05 ± 3.79	4.44 ± 4.46	3.60 ± 2.45	3.62 ± 3.41	3.81 ± 3.00	0.39
pH	7.37 ± 0.12	7.38 ± 0.11	7.37 ± 0.10	7.38 ± 0.13	7.34 ± 0.13	< 0.05
PaCO2 (mmHg)	39.04 ± 15.43	40.26 ± 13.80	40.27 ± 23.99	37.42 ± 12.87	41.06 ± 14.92	0.15
PaO2 (mmHg)	106.61 ± 64.52	113.22 ± 80.55	104.33 ± 55.00	107.47 ± 56.74	91.83 ± 52.71	0.08
TCO2 (mEq/L)	21.85 ± 5.76	20.38 ± 4.40	22.19 ± 5.25	22.58 ± 6.60	21.91 ± 5.37	< 0.01
Number of admissions with outcomes (n)						
IHCA/1000 adm	0.90 (50)	1.71 (15)	0.71 (13)	1.66 (15)	0.36 (7)	–
UIT/1000 adm	6.44 (355)	12.67 (111)	5.32 (97)	7.09 (64)	4.34 (83)	–
Borderline/1000 adm	15.70 (865)	23.41 (205)	12.29 (224)	26.05 (235)	10.52 (201)	–
DNR suggestion/1000 adm	1.01 (56)	2.51 (22)	0.49 (9)	2.32 (21)	0.20 (4)	–

### Key aspect 1: Predictive performance

**Fig. 1:**
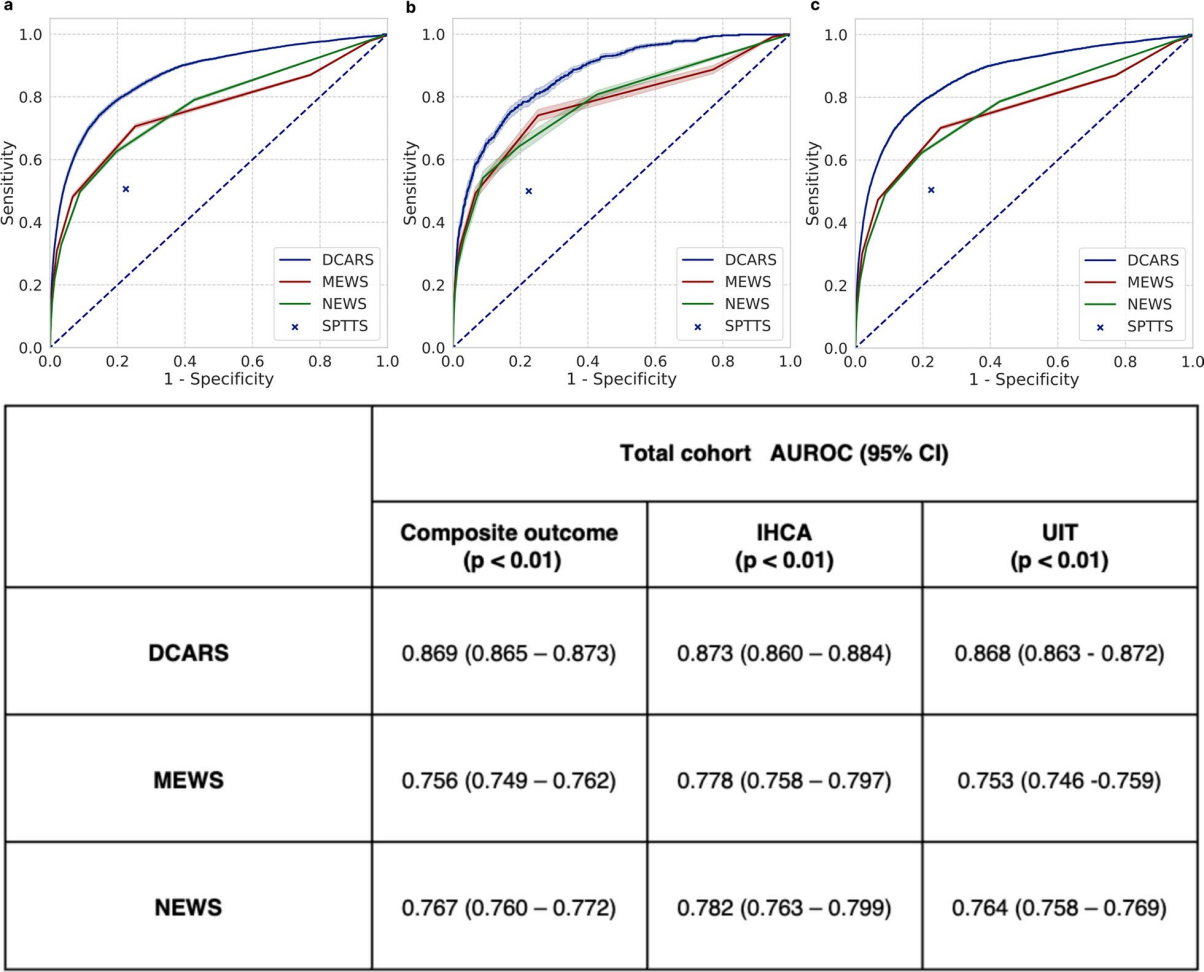
The performance of each model predicting in-hospital cardiac arrest or unplanned intensive care unit transfer. **a** The ROC curve for predicting deterioration (IHCA or UIT). **b** The ROC curve for predicting IHCA. **c** The ROC curve for predicting UIT. DCARS: deep learning-based cardiac arrest risk score; MEWS: Modified Early Warning Score; NEWS: National Early Warning Score; SPTTS: single-parameter track-and-trigger system; AUROC: area under the receiver operating characteristic curve; ROC: receiver operating characteristic curve; CI: confidence interval; IHCA: in-hospital cardiac arrest; UIT: unplanned intensive care unit transfer

As shown in Fig. 1, the DeepCARS™ outperformed conventional triggering systems in predicting composite primary outcomes (AUROC: 0.869 DeepCARS™ vs. 0.756 MEWS/0.767 NEWS). When comparing the sensitivity of composite outcome prediction at the same specificity level as conventional systems, the DeepCARS™ outperformed the MEWS, NEWS, and SPTTS at every specificity level (Additional file 1: Table S1).

Additionally, we evaluated how predictive performance changed over time before the primary event. The performance of the DeepCARS™, MEWS, and NEWS increased as the primary event (time zero) approached; however, the DeepCARS™ maintained superior performance across all time points, with performance saturating at a prediction time of 3 h before the event (Additional file 1: Fig. S2).

### Key aspect 2: Alarm performance

**Fig. 2:**
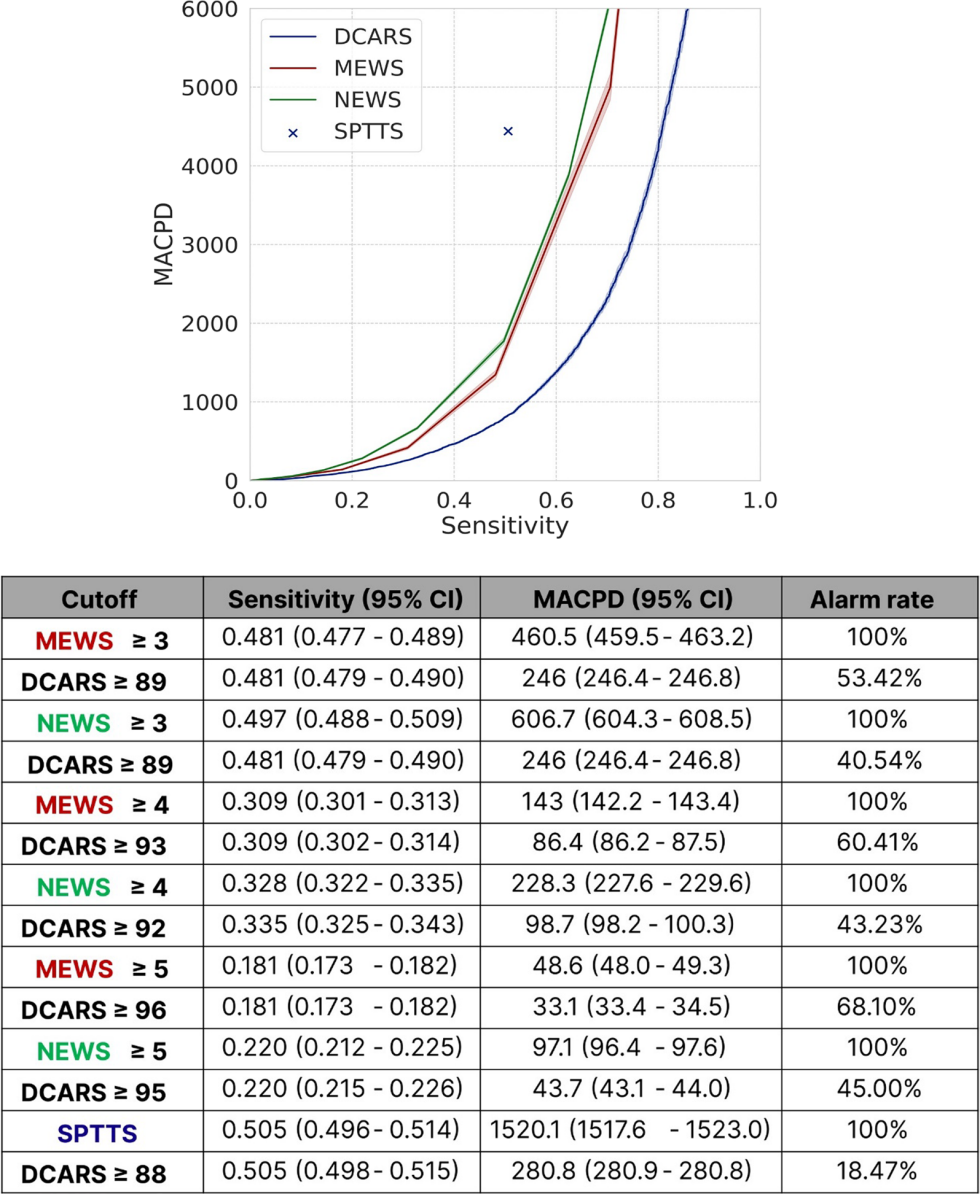
Comparison of mean alarm counts/day/1,000 beds at the same sensitivity level for each prediction model. MACPD: mean alarm counts per day per 1,000 beds; DCARS, deep learning-based cardiac arrest risk score; MEWS: modified early warning score; NEWS: national early warning score; SPTTS: single-parameter track-and-trigger system

**Fig. 3:**
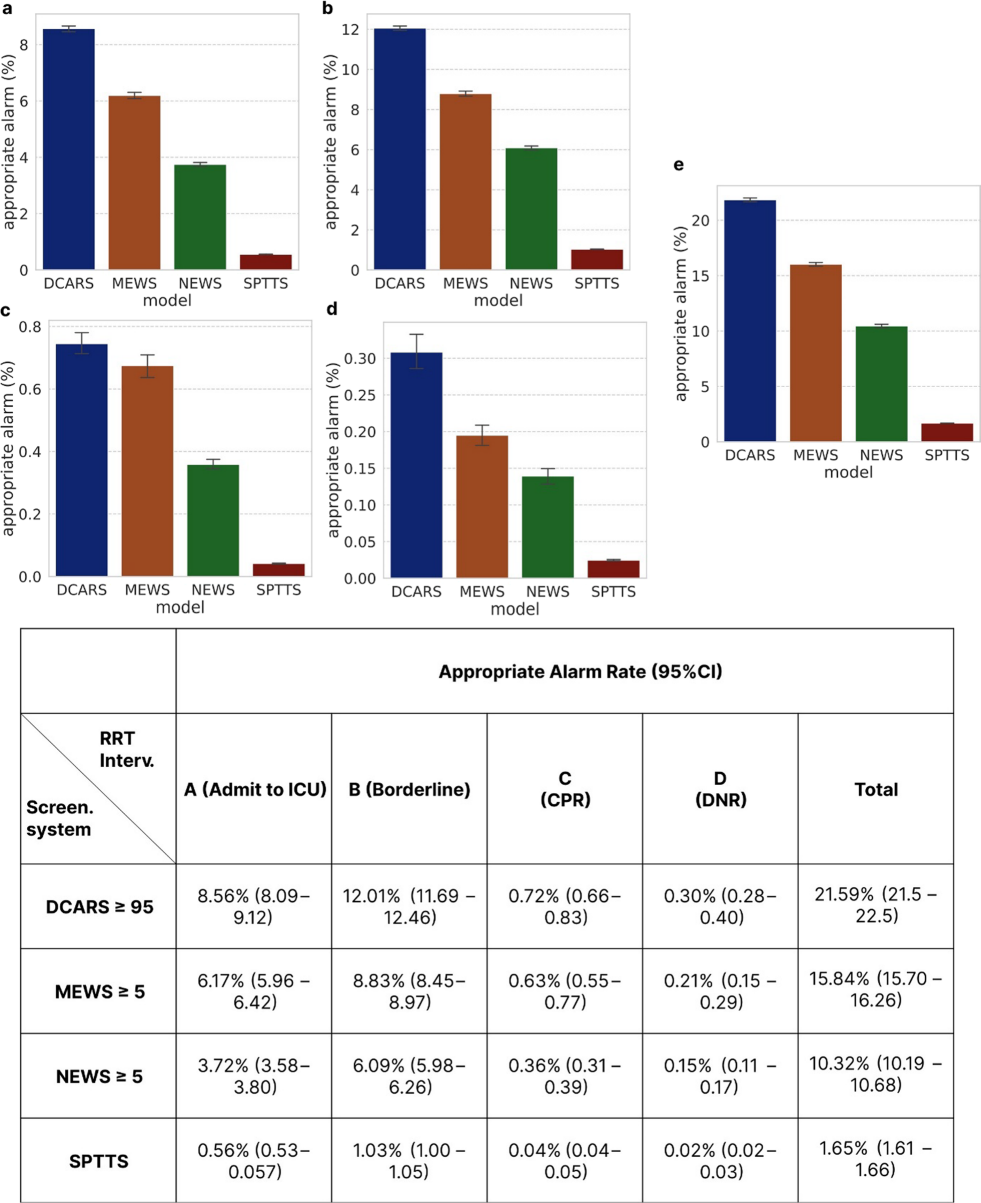
The comparison of appropriate alarm rates at the same sensitivity level for each prediction model. DCARS: deep learning-based cardiac arrest risk score; MEWS: Modified Early Warning Score; NEWS: National Early Warning Score; SPTTS: single-parameter track-and-trigger system; UIT: unplanned intensive care unit transfer; DNR: do not resuscitate; Screen.: Screening; Interv.: Intervention

The DeepCARS™ resulted in a significant reduction in MACPD, compared with conventional methods at the same sensitivity level (Fig. 2). Specifically, assuming a 100% alarm rate for the SPTTS, the alarm rate of the DeepCARS™ was reduced to 18.47%, representing an improvement of 441.4%. Additionally, when compared with the MEWS and NEWS, the alarm rates were reduced to 53.42% and 31.25%, respectively. Regarding alarm appropriateness (Fig. 3), alarms generated by the DeepCARS™ resulted in more clinical interventions by the RRT (21.59%), compare with the MEWS (15.84%), NEWS (10.32%), and SPTTS (1.65%). The SPTTS not only yielded the lowest rate of appropriate alarms, but the absolute value itself was extremely low, indicating that the SPTTS produced more false than true alarms.

### Key aspect 3: timeliness

**Fig. 4:**
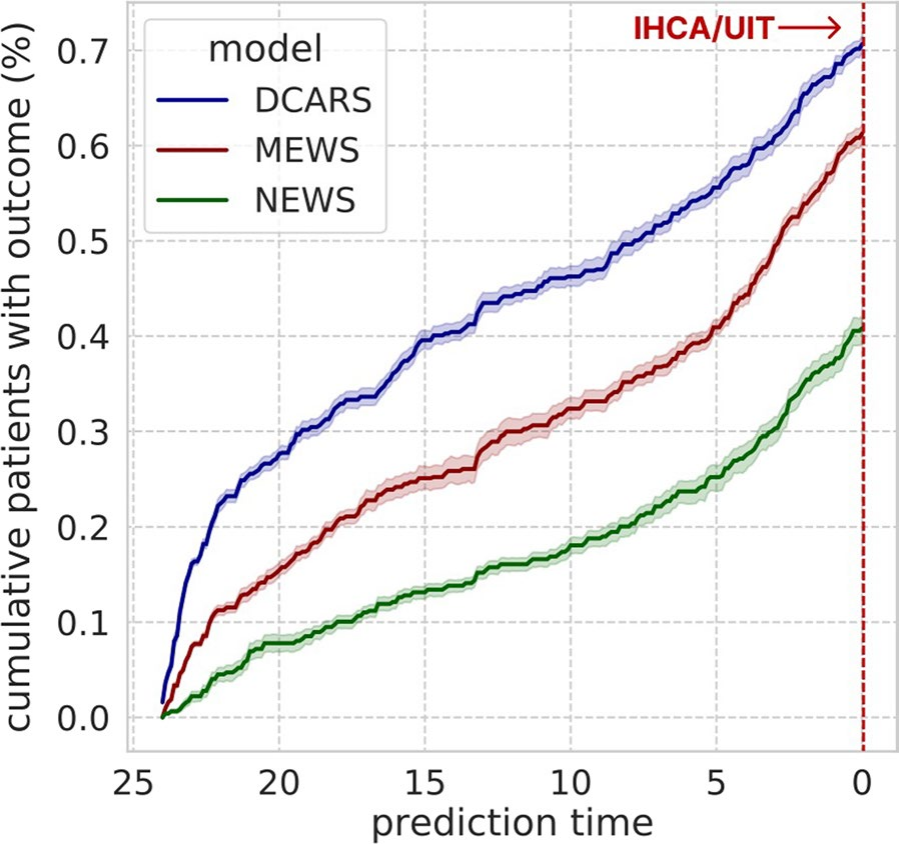
The cumulative percentage of patients with IHCA and UIT for each prediction model. DCARS: deep learning-based cardiac arrest risk score; MEWS: modified early warning score; NEWS: national early warning score; UIT: unplanned intensive care unit transfer; IHCA: in-hospital cardiac arrest

The DeepCARS™ also provided more timely predictions than did the MEWS and NEWS based on the cumulative percentage of detected events within 24 h to 30 min before the primary event (Fig. 4). Specifically, 15 h before deterioration, the cumulative percentage of patients identified by the DeepCARS™ was 38.7%, whereas these rates were 25.2% and 26.5% for the MEWS and NEWS, respectively.

### Key aspect 4: subgroup analysis

**Fig. 5:**
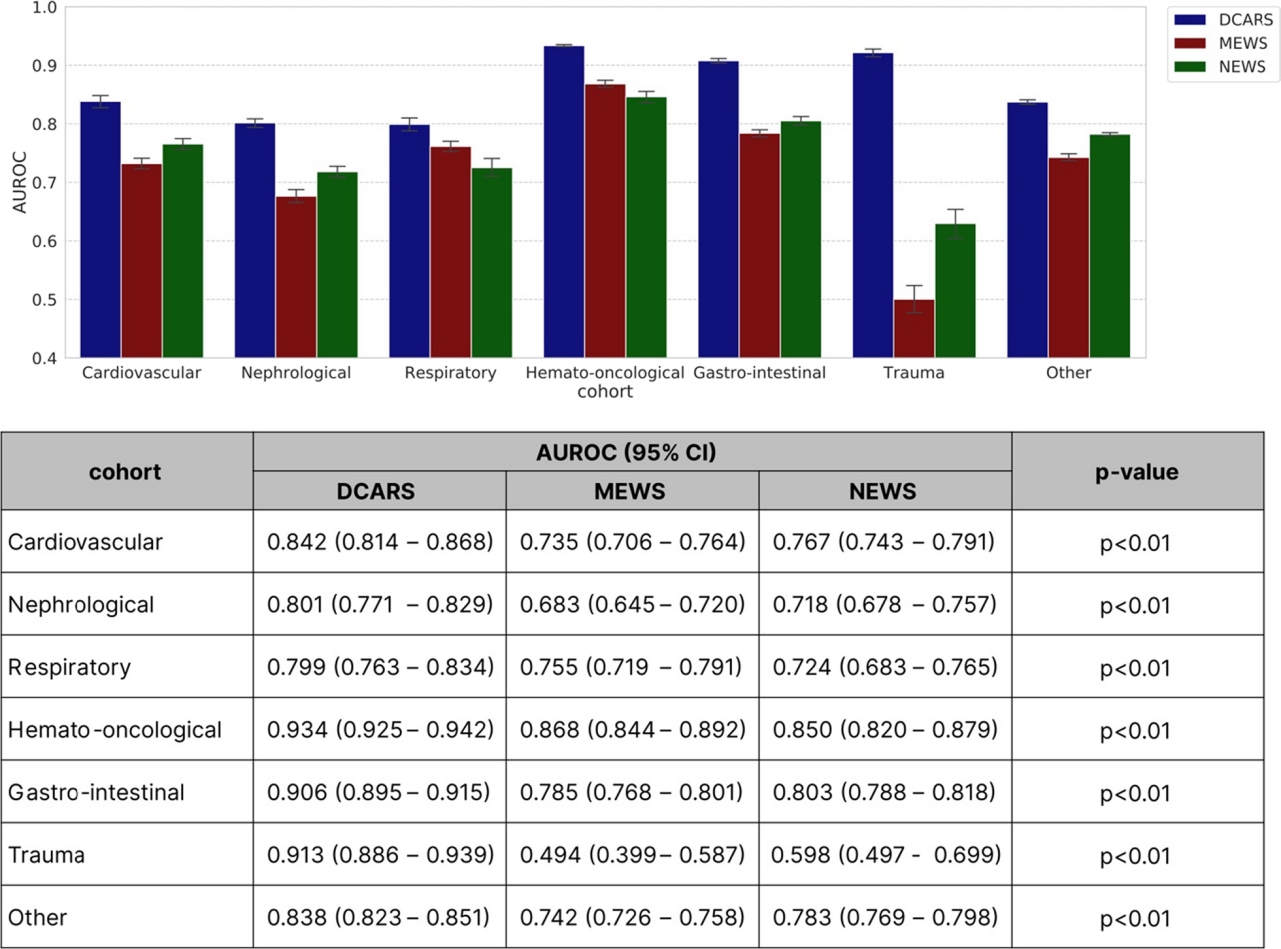
The performance of each model predicting IHCA and UIT in different cohorts. AUROC: area under the receiver operating characteristic curve; DCARS: deep learning-based cardiac arrest risk score; MEWS: Modified Early Warning Score; NEWS: National Early Warning Score; UIT: unplanned intensive care unit transfer; IHCA: in-hospital cardiac arrest

As shown in Fig. 5, the DeepCARS™ achieved a higher predictive performance for IHCA and UIT in each department. The superiority of the DeepCARS™ was maintained regardless of the department of admission. The DeepCARS™ had the highest predictive performance (AUROC: 0.934), especially in patients with hemato-oncological disease. Model performance was also consistent across age groups, sexes, hospitals, and surgical status (Additional file 1: Fig. S3).

### Model calibration

The DeepCARS™ was well calibrated, compared with conventional methods (Additional file 1: Fig. S4), and it yielded a lower average absolute error between the outcome and estimated probabilities than that of conventional methods (0.181 vs. 0.335/0.326).

## Discussion

Our study indicated that the predictive performance of the DeepCARS™ for IHCA or UIT was superior to that of the MEWS, NEWS, and SPTTS in patients admitted to general wards. At the same sensitivity level, the total alarm count was significantly reduced using the DeepCARS™, which also increased the relative number of appropriate alarms leading to real activation of RRT interventions. In addition, the DeepCARS™ predicted the outcomes of patients earlier, and its predictive performance remained superior to that of conventional methods, regardless of department of admission, patient age, sex, hospital, or surgical status. Therefore, better predictions with fewer alarm counts and earlier predictions indicate that the DeepCARS™ is an effective alternative screening tool to conventional triggering systems for the RRS.

The main strength of our study was that we clearly distinguished true alarms that led to actual RRT interventions from all alarms in a prospective manner. To our knowledge, this is the first study to prospectively collect and triage each alarm system for RRT intervention. In our study, borderline interventions included fluid therapy, prescription of antibiotics or other medications, oxygen therapy, and recommendation for further specific evaluation by the RRT. Although these interventions are not as dramatic as UIT or IHCA, they account for the majority of RRT actions and improve clinical course, thereby helping to avoid potentially severe outcomes [1, 17]. By defining borderline interventions and analyzing them according to alarms, we were able to calculate the exact number of appropriate alarms placing patients at risk of IHCA or UIT. In addition, DNR recommendations by the RRT are relatively common in clinical practice, such as in patients with terminal cancer or no further possibility of resuscitation [18, 19]. However, a retrospective study design can make it difficult to identify and tag which alarms are associated with borderline interventions or DNR suggestions by the RRT. Our prospective study design enabled a more accurate validation by preventing the misclassification of appropriate alarms, providing stronger evidence of the clinical practicality and efficacy of the DeepCARS™.

Numerous studies have developed machine learning-based algorithms for predicting IHCA [7, 8, 20–24]. Churpek et al. revealed that the random forest algorithm was more accurate than the MEWS in predicting IHCA, ICU admission, and death in wards for patients who experienced attempted resuscitation [20]. The Mayo Clinic EWS and electronic cardiac arrest risk triage score also exhibited better performance in predicting IHCA or ICU transfer than did the NEWS [23, 25]. These algorithms rely on a large number of variables and require complex calculations based on a combination of demographics, vital signs, and laboratory test results. Therefore, lack of demographic data and time lags between events and laboratory tests can lower their predictive performance and make them difficult to apply in real-world settings. In 2022, a time-series early warning score (TEWS) for predicting IHCA using only basic vital signs was validated [21]. The predictive performance of the TEWS for IHCA was superior to that of the MEWS. The TEWS and DeepCARS™ differ in several aspects, including their model architectures, training methods, preprocessing methods, and exclusion criteria. The main differences between them are their inputs and outputs: while the DeepCARS™ uses age and recorded time as predictor variables for predicting cardiac arrest within 24 h in addition to vital signs, the TEWS focuses solely on vital signs to predict cardiac arrest within 48 h. Age was added as a predictor variable to the DeepCARS™ to provide basic patient information for the model to cluster patients according to age and vital signs. Age is important because vital signs associations can differ by age group. Additionally, the recorded time provides critical information regarding the length of stay and monitoring intensity, providing greater insight into the severity of the patient’s condition, compared with vital sign values alone. Finally, the DeepCARS™ is more advantageous than the TEWS, given that the latter was developed and validated in a single-center retrospective study.

Delays in RRS initiation and ICU transfer have been associated with increased mortality and morbidity [26]. Although vital signs are usually monitored continuously in the ICU, nurses in general wards measure vital signs three or four times daily. Thus, early detection of clinical deterioration by EWS and suitable interventions for RRT are crucial for patient prognosis [27, 28]. In our study, the DeepCARS™ provided more time to intervene, compared with the other traditional triggering systems. In addition, DeepCARS™ performance was sustained regardless of department of admission, age, sex, hospital, or surgical status. The current results indicate that the DeepCARS™ may be superior to or at least not inferior to conventional triggering systems in the RRS, highlighting its potential as an effective system for screening high-risk patients in general wards.

This study had some limitations. First, we did not examine the relationship between RRS activation by the DeepCARS™ and IHCA reduction. Although alarms triggered by the DeepCARS™ led to more adequate RRT interventions, compared with those triggered by other methods, the study period was too short for the evaluation of long-term prognosis. Second, we did not evaluate the appropriateness of every RRT intervention, as we assumed that the detection of clinical deterioration by the EWS would result in appropriate intervention. However, in real-world clinical practice, the judgment of the RRT may influence the decision to intervene and the quality of the intervention. Therefore, guidelines for appropriate standard interventions should be developed and verified. Third, selection bias may have occurred given that all hospitals included in this study had university affiliations. In addition, all four hospitals have mature RRS, and it is necessary to evaluate DeepCARS™ performance in hospitals that have recently implemented RRS and those without an established RRS, as the incidence and reduction of IHCA may depend on the maturity of the RRS. Finally, the DeepCARS™ was evaluated only in South Korea, necessitating further studies among other ethnic groups.

## Conclusions

The current study demonstrates that the DeepCARS™, an AI-based tool utilizing deep learning and vital sign data, outperforms conventional early warning scores such as the MEWS, NEWS, and SPTTS in accurately predicting IHCA or UIT. Our data also suggest that the DeepCARS™ produces appropriate alarms that lead to timely RRT intervention, highlighting its potential as an effective screening tool for detecting clinical deterioration in hospitalized patients. However, further clinical trials are required to assess the impact of the DeepCARS™ on patient outcomes and evaluate its feasibility for clinical implementation.

### Supplementary Information

**Additional file 1: Table S1.** Comparison of performance for prediction of composite outcome at the same specificity. DCARS, Deep learning-based cardiac arrest risk score; MEWS, modified early warning score; NEWS, national early warning score; Sen, sensitivity; Spec, specificity; PPV, positive predictive value; LR, likelihood ratio; NPV, negative predictive value; NNE, number needed to examine; F1-score, harmonic mean of the precision and recall. **Fig. S1** Flow diagram for the prospective multicenter cohort study in four referral hospitals in South Korea. IHCA: in-hospital cardiac arrest; UIT: unplanned intensive care unit transfer; DNR: do not resuscitate. **Fig. S2** Prediction model performance for timeline 24 h–0.5 h before IHCA or UIT. IHCA: in-hospital cardiac arrest; UIT: unplanned intensive care unit transfer; DCARS: deep learning-based cardiac arrest risk score; MEWS: Modified Early Warning Score; NEWS: National Early Warning Score. **Fig. S3** Subgroup analysis of prediction model performance by age group, sex, hospital, and cohort. a. Subgroup analysis by age group. b. Subgroup analysis by sex. c. Subgroup analysis by hospital. d. Subgroup analysis by cohort. AUROC: area under the receiver operating characteristic curve; DCARS: deep learning-based cardiac arrest risk score; MEWS: Modified Early Warning Score; NEWS: National Early Warning Score. **Fig. S4** Calibration plots for each prediction model. DCARS: deep learning-based cardiac arrest risk score; MEWS: Modified Early Warning Score; NEWS: National Early Warning Score

## Data Availability

Completely de-identified participant data as well as full dataset will be shared upon reasonable request to the corresponding author, after approval by the scientific steering committee of this study group. Consent was not obtained, but the presented data are anonymized, and the risk of identification is low.

## Abbreviations

IHCA: In-hospital cardiac arrest
RRT: Rapid response team
RRS: Rapid response system
DCARS: Deep learning-based cardiac arrest risk score
MEWS: Modified Early Warning Score
NEWS: National Early Warning Score
SPTTS: Single-parameter track-and-trigger system
AUROC: Area under the receiver operating characteristic curve
ROC: Receiver operating characteristic curve
CI: Confidence interval
DNR: Do not resuscitate
UIT: Unplanned intensive care unit transfer
ICU: Intensive care unit
