# Supplementary material for: Prospective, multicenter validation of the deep learning-based cardiac arrest risk management system for predicting in-hospital cardiac arrest or unplanned intensive care unit transfer in patients admitted to general wards
Source: Crit Care. 2023 Sep 5;27:346. doi: 10.1186/s13054-023-04609-0 (PMC10481524; doi:10.1186/s13054-023-04609-0)

***Additional file 1***

**The prospective multicenter validation of a deep-learning based cardiac arrest risk score for predicting in-hospital cardiac arrest or unplanned intensive care unit transfer in patients admitted to general wards**

**Table S1.** Comparison of performance for prediction of composite outcome at the same specificity

| **Cutoff** | **sen** | **spec** | **PPV** | **NPV** | **Positive**  **LR** | **Negative**  **LR** | **NNE** | **F1-score** | **NRI** |
| --- | --- | --- | --- | --- | --- | --- | --- | --- | --- |
| MEWS ≥ 2 | 0.706 (0.698 - 0.713) | 0.747 (0.746 - 0.747) | 0.0081 (0.0080 - 0.0083) | 0.998 (0.998 - 0.999) | 2.790 (2.748 - 2.818) | 0.393 (0.404 - 0.384) | 123.4 (125.0 - 120.4) | 0.0160 (0.0158 - 0.0164) |  |
| DCARS ≥ 67 | 0.827 (0.821 - 0.833) | 0.747 (0.746 - 0.747) | 0.0094 (0.0091 - 0.0096) | 0.999 (0.999 - 0.999) | 3.268 (3.232 - 3.292) | 0.231 (0.239 - 0.223) | 106.3 (109.8 - 104.1) | 0.0185 (0.0180 - 0.0189) | 0.01253 (0.01132 - 0.01382) |
| MEWS ≥ 3 | 0.481 (0.477 - 0.489) | 0.933 (0.932 - 0.933) | 0.0205 (0.0201 - 0.0209) | 0.998 (0.998 - 0.999) | 7.179 (7.014 - 7.298) | 0.556 (0.561 - 0.547) | 48.7 (49.7 - 47.8) | 0.0393 (0.0385 - 0.0400) |  |
| DCARS ≥ 84 | 0.598 (0.590 - 0.603) | 0.933 (0.932 - 0.933) | 0.0251 (0.0245 - 0.0257) | 0.998 (0.998 - 0.998) | 8.925 (8.676 - 9.000) | 0.430 (0.439 - 0.425) | 39.8 (40.8 - 38.9) | 0.0480 (0.0470 - 0.0492) | 0.00761 (0.00724 - 0.00798) |
| MEWS ≥ 4 | 0.309 (0.301 - 0.313) | 0.980 (0.979 - 0.980) | 0.0424 (0.0414 - 0.0432) | 0.998 (0.998 - 0.998) | 15.449 (14.333 - 15.649) | 0.705 (0.713 - 0.701) | 23.5 (24.1 - 23.1) | 0.0745 (0.0727 - 0.0759) |  |
| DCARS ≥ 91 | 0.378 (0.372 - 0.383) | 0.980 (0.979 - 0.980) | 0.0519 (0.0508 - 0.0528) | 0.998 (0.998 - 0.998) | 18.899 (17.714 - 19.149) | 0.634 (0.641 - 0.629) | 19.2 (19.6 - 18.9) | 0.0912 (0.0893 - 0.0927) | 0.00969 ().00913 - 0.01025) |
| MEWS ≥ 5 | 0.181 (0.173 - 0.182) | 0.993 (0.993 - 0.993) | 0.0731 (0.0701 - 0.0752) | 0.997 (0.997 - 0.998) | 25.857 (24.714 - 25.999) | 0.824 (0.832 - 0.823) | 13.6 (14.2 - 13.2) | 0.1041 (0.0997 - 0.1064) |  |
| DCARS ≥ 94 | 0.248 (0.243 - 0.254) | 0.993 (0.993 - 0.993) | 0.0835 (0.0808 - 0.0853) | 0.998 (0.997 - 0.998) | 35.428 (34.714 - 36.284) | 0.757 (0.762 - 0.751) | 11.9 (12.3 - 11.7) | 0.1249 (0.1212 - 0.1277) | 0.02595 (0.02132 - 0.03058) |
| MEWS ≥ 6 | 0.084 (0.080 - 0.089) | 0.997 (0.997 - 0.997) | 0.0895 (0.0869 - 0.0948) | 0.997 (0.997 - 0.997) | 27.999 (26.666 - 29.666) | 0.918 (0.922 - 0.913) | 11.1 (11.5 - 10.5) | 0.0866 (0.0833 - 0.0918) |  |
| DCARS ≥ 96 | 0.181 (0.173 - 0.182) | 0.997 (0.997 - 0.997) | 0.1133 (0.1084 - 0.1204) | 0.997 (0.997 - 0.997) | 60.333 (57.666 - 60.666) | 0.821 (0.829 - 0.820) | 8.8 (9.2 - 8.3) | 0.1393 (0.1332 - 0.1449) | 0.08766 (0.08421 - 0.09111) |
| NEWS ≥ 2 | 0.625 (0.613 - 0.631) | 0.803 (0.803 - 0.804) | 0.0092 (0.0089 - 0.0093) | 0.998 (0.998 - 0.998) | 3.172 (3.111 - 3.219) | 0.466 (0.481 - 0.458) | 108.6 (112.3 - 107.5) | 0.0181 (0.0175 - 0.0183) |  |
| DCARS ≥ 72 | 0.788 (0.784 - 0.796) | 0.803 (0.802 - 0.803) | 0.0115 (0.0113 - 0.0117) | 0.999 (0.999 - 0.999) | 4.000 (3.959 - 4.040) | 0.264 (0.269 - 0.254) | 86.9 (88.4 - 85.4) | 0.0225 (0.0222 - 0.0230) | 0.00132 (0.00088 - 0.00240) |
| NEWS ≥ 3 | 0.497 (0.488 - 0.509) | 0.911 (0.910 - 0.911) | 0.0161 (0.0157 - 0.0164) | 0.998 (0.998 - 0.998) | 5.584 (5.422 - 5.719) | 0.552 (0.562 - 0.538) | 62.1 (63.6 - 60.9) | 0.0311 (0.0304 - 0.0317) |  |
| DCARS ≥ 82 | 0.650 (0.644 - 0.656) | 0.911 (0.910 - 0.911) | 0.0207 (0.0202 - 0.0210) | 0.999 (0.998 - 0.999) | 7.303 (7.155 - 7.370) | 0.384 (0.391 - 0.377) | 48.3 (49.5 - 47.6) | 0.0400 (0.0391 - 0.0406) | 0.00457 (0.00415 - 0.00491) |
| NEWS ≥ 4 | 0.328 (0.322 - 0.335) | 0.967 (0.966 - 0.967) | 0.0282 (0.0278 - 0.0289) | 0.998 (0.997 - 0.998) | 9.939 (9.47 - 10.151) | 0.694 (0.701 - 0.687) | 35.4 (35.9 - 34.6) | 0.0519 (0.0511 - 0.0532) |  |
| DCARS ≥ 89 | 0.481 (0.479 - 0.490) | 0.967 (0.966 - 0.967) | 0.0390 (0.0377 - 0.0398) | 0.998 (0.998 - 0.998) | 14.575 (14.088 - 14.848) | 0.536 (0.539 - 0.527) | 25.6 (26.5 - 25.1) | 0.0721 (0.0698 - 0.0736) | 0.01180 (0.01016 - 0.01257) |
| NEWS ≥ 5 | 0.220 (0.212 - 0.225) | 0.986 (0.986 - 0.986) | 0.0445 (0.0434 - 0.0452) | 0.997 (0.997 - 0.997) | 15.714 (15.142 - 16.071) | 0.791 (0.799 - 0.786) | 22.4 (23.0 - 22.1) | 0.0740 (0.0720 - 0.0752) |  |
| DCARS ≥ 92 | 0.335 (0.325 - 0.343) | 0.986 (0.986 - 0.986) | 0.0614 (0.0592 - 0.0626) | 0.998 (0.998 - 0.998) | 23.928 (23.214 - 24.499) | 0.674 (0.684 - 0.666) | 16.2 (16.8 - 15.9) | 0.1037 (0.1001 - 0.1058) | 0.02208 (0.01729 - 0.02431) |
| NEWS ≥ 6 | 0.144 (0.140 - 0.150) | 0.994 (0.993 - 0.994) | 0.0618 (0.0606 - 0.0643) | 0.997 (0.997 - 0.997) | 23.999 (19.999 - 24.999) | 0.861 (0.866 - 0.855) | 16.1 (16.5 - 15.5) | 0.0864 (0.0845 - 0.0900) |  |
| DCARS ≥ 95 | 0.220 (0.215 - 0.226) | 0.994 (0.993 - 0.994) | 0.0897 (0.0888 - 0.0926) | 0.997 (0.997 - 0.997) | 36.666 (30.714 - 37.665) | 0.784 (0.79 - 0.778) | 11.1 (11.2 - 10.7) | 0.1274 (0.1256 - 0.1313) | 0.03869 (0.03445 - 0.04438) |
| SPTTS | 0.505 (0.496 - 0.514) | 0.775 (0.774 - 0.775) | 0.0065 (0.0064 - 0.0067) | 0.998 (0.998 - 0.998) | 2.244 (2.194 - 2.283) | 0.638 (0.651 - 0.627) | 153.8 (156.2 - 149.2) | 0.0128 (0.0126 - 0.0132) |  |
| DCARS ≥ 69 | 0.807 (0.792 - 0.812) | 0.775 (0.774 - 0.775) | 0.0103 (0.0101 - 0.0104) | 0.999 (0.999 - 0.999) | 3.586 (3.504 - 3.608) | 0.249 (0.268 - 0.242) | 97.0 (99.0 - 96.1) | 0.0203 (0.0199 - 0.0205 | 0.01013 (0.00983 - 0.01043) |

DCARS, Deep learning-based cardiac arrest risk score; MEWS, modified early warning score; NEWS, national early warning score; Sen, sensitivity; Spec, specificity; PPV, positive predictive value; LR, likelihood ratio; NPV, negative predictive value; NNE, number needed to examine; F1-score, harmonic mean of the precision and recall

**Fig. S1** Flow diagram for the prospective multicenter cohort study in four referral hospitals in South Korea

**Fig. S2** Prediction model performance for timeline 24 hours–0.5 hours before IHCA or UIT

**
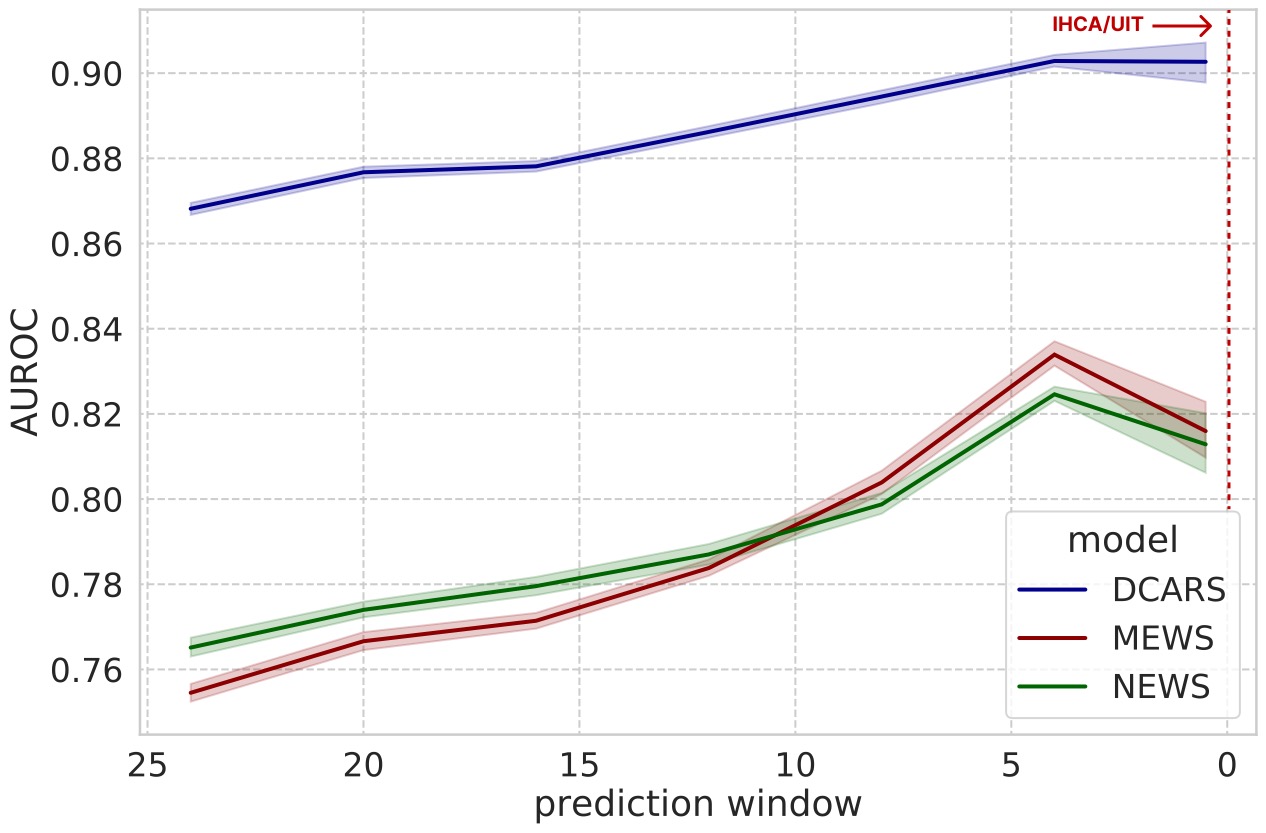
**

**Fig. S3** Subgroup analysis of prediction model performance by age group, sex, hospital, and cohort

**
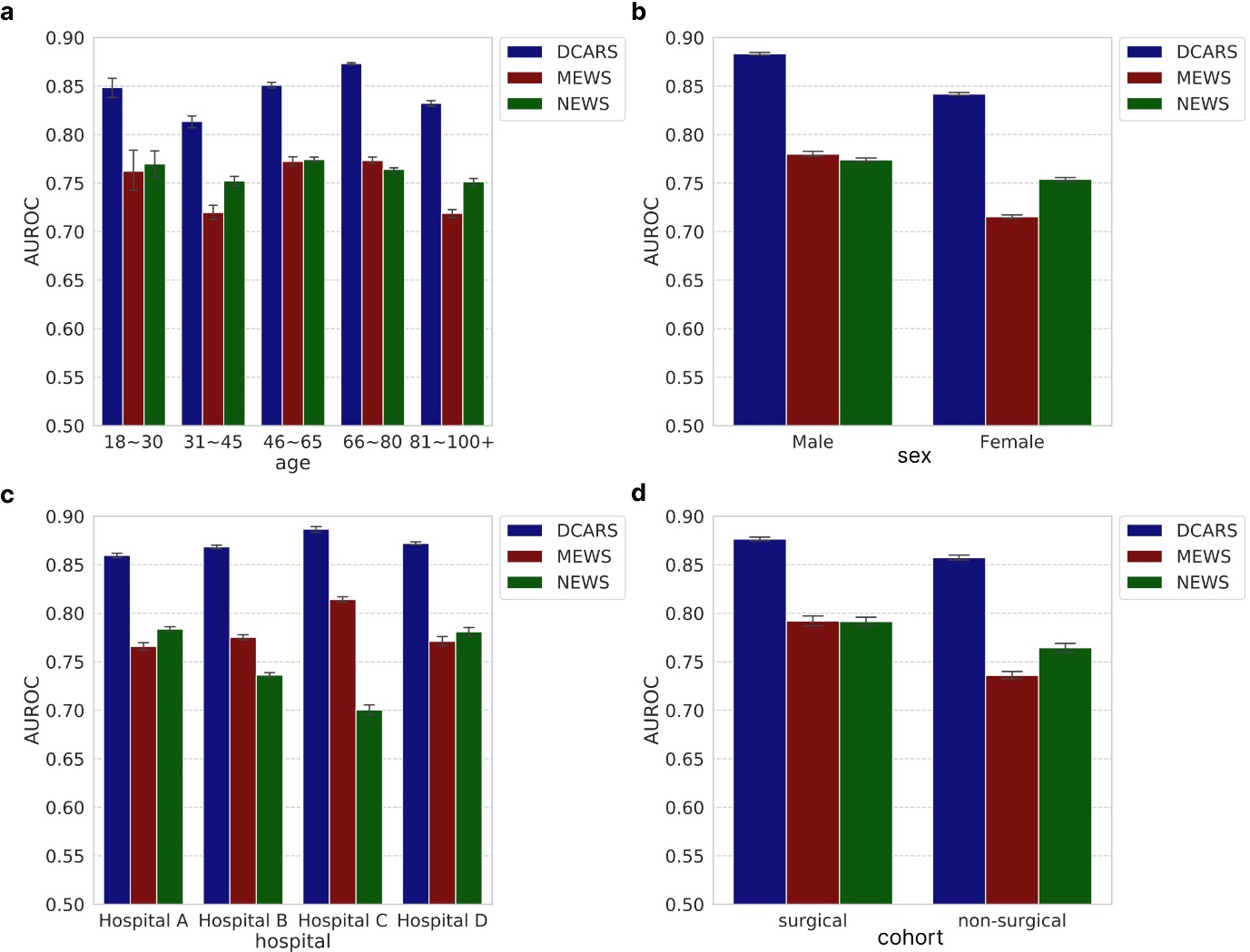
**

**Fig. S4** Calibration plots for each prediction model


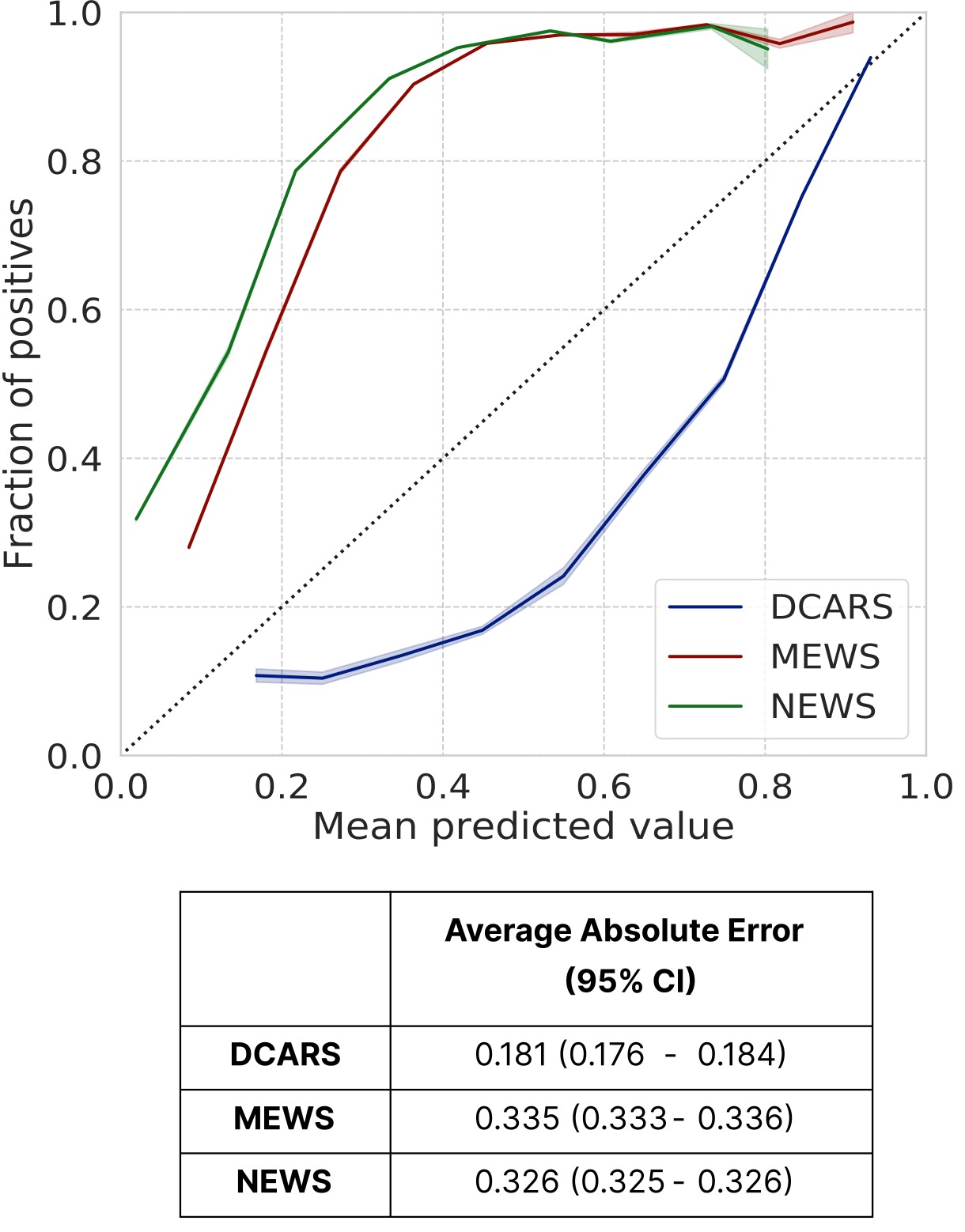

Supplement: Supplementary file 1 — Additional file 1: Table S1. Comparison of performance for prediction of composite outcome at the same specificity. DCARS, Deep learning-based cardiac arrest risk score; MEWS, modified early warning score; NEWS, national early warning score; Sen, sensitivity; Spec, specificity; PPV, positive predictive value; LR, likelihood ratio; NPV, negative predictive value; NNE, number needed to examine; F1-score, harmonic mean of the precision and recall. Fig. S1 Flow diagram for the prospective multicenter cohort study in four referral hospitals in South Korea. IHCA: in-hospital cardiac arrest; UIT: unplanned intensive care unit transfer; DNR: do not resuscitate. Fig. S2 Prediction model performance for timeline 24 h–0.5 h before IHCA or UIT. IHCA: in-hospital cardiac arrest; UIT: unplanned intensive care unit transfer; DCARS: deep learning-based cardiac arrest risk score; MEWS: Modified Early Warning Score; NEWS: National Early Warning Score. Fig. S3 Subgroup analysis of prediction model performance by age group, sex, hospital, and cohort. a. Subgroup analysis by age group. b. Subgroup analysis by sex. c. Subgroup analysis by hospital. d. Subgroup analysis by cohort. AUROC: area under the receiver operating characteristic curve; DCARS: deep learning-based cardiac arrest risk score; MEWS: Modified Early Warning Score; NEWS: National Early Warning Score. Fig. S4 Calibration plots for each prediction model. DCARS: deep learning-based cardiac arrest risk score; MEWS: Modified Early Warning Score; NEWS: National Early Warning Score [file 13054_2023_4609_MOESM1_ESM.docx]
